# Government-Guaranteed Sera: A National Need

**Published:** 1912-06-01

**Authors:** 


### GOVERNMENT-GUARANTEED SERA: A NATIONAL NEED.

In these days when there is a tendency on the part of the public to look for government protection in nearly everything, from table meat to certification, it is astonishing that no one has yet suggested that it is the duty of the State to see that every flask of serum sold should be properly prepared, and, as far as human effort can make it so, efficient. Yet this is a guarantee which every government might reasonably be expected to give. At present it is free to any individual or to any firm to manufacture sera and to sell these to the public or to the profession at whatever price may be obtainable. Although the argument is obvious that no individual or firm will be able for long to sell a worthless brand of serum, practical experience shows that this trial by results does not work well. It is true again that some firms stamp every flask or tube of serum sent out with the name and date, and that these firms, generally, are willing to exchange old tubes for new with almost the same nonchalance and generosity that the old beggar displayed when he gave a bright new lamp for Aladdin's old rusted thing of power. But all firms are not equally careful or equally generous, and it is not uncommon to purchase, especially when the consumer is in a hurry, an old brand of serum which is inert and useless, and to find, on subsequent expostulation, that the only compensation offered is the stereotyped formula "We are unable to take back serum once sold." Serum therapy is yet in its embryonic stage, and the use of serum as a remedy for ordinary complaints such as coryza or diarrhœa is not general.

The Government has already faced the state of affairs engendered by the knowledge on the part of the layman that aspirin is a good remedy in cases of rheumatism, and ammoniated quinine in cases of a commencing cold. It requires that the tablets sold across the chemist's counter shall be really composed of aceto-salicylic acid, and that the nauseating white fluid in the bottle labelled ammoniated quinine shall satisfy certain conditions laid down in the Pharmacopœia. On occasion it shows that this interest in the purity of the drugs supplied to the public is not fictitious by prosecuting some chemist or drug vendor. But it wholly neglects sera. Among the most potent poisons that can be enumerated, certain vaccines and sera are not scheduled: anyone can buy them, and presumably, the safety of the public is guarded by the fact that few outside the rank of the medical profession know what they are, how they can be used, or where they can be procured. All the sera are drugs in a sense: it is as important that they should be pure, and, again with the proviso as far as is humanly possible, efficacious. At present the purchaser has no guarantee when he buys them that he is buying anything which will have an effect even remotely similar to that which it is expected to have. He may buy pure water, or water rendered slightly turbid and gumlike by the addition of a small quantity of gelatine solution, and confidently swallow it under the impression that he is imbibing an anti-b.c.c. serum. He has no means of testing it, even of knowing if it be serum at all. His only guarantee, of course, is to obtain it from a reliable firm; and so long as serum is only purchased by the doctors who are able to gauge the reliability of firms that advertise their serum products, all is doubtless very well. But when serum treatment becomes a home method of curing disease (as it threatens to become), it is necessary that the Government should step in and look after the interests of the serum-buying public just as now it looks after the interests of the aspirin-buying public. Every tube of serum should at least be required to be stamped and dated, and it would be highly desirable to have some Government mark affixed whereby it is possible at a glance to tell the excellence of the product. Such excellence can of course only be guaranteed relatively, but even a provisional guarantee is something to be grateful for when the absence of any guarantee whatsoever puts it in the power of any enterprising quack to cheat the public in a wholesale manner by vending sera which are not sera at all.

